# Therapeutic anti-inflammatory immune potentials of some seaweeds extracts on chemically induced liver injury in mice

**DOI:** 10.1038/s41598-025-87379-9

**Published:** 2025-02-05

**Authors:** Eman Bases, Mostafa M. El-Sheekh, Shimaa M. El Shafay, Rania El-shenody, Mohamed Nassef

**Affiliations:** 1 Botany Department, Faculty of Science, Tanta University, Tanta, Egypt; 2 Zoology Department, Faculty of Science, Tanta University, Tanta, Egypt

**Keywords:** Seaweeds, Immunomodulatory, Anti-inflammatory, Antioxidants, Hepatoprotective, Immunology, Microbiology, Plant sciences

## Abstract

Carbon tetrachloride (CCl_4_) is a well-known hepatotoxin. This work aimed to assess the therapeutic anti-inflammatory immune potentials of the seaweeds *Padina pavonia* and *Jania rubens* extracts on carbon tetrachloride (CCL_4_)-caused liver damage in mice. Our experimentation included two testing regimens: pre-treatment and post-treatment of *P. pavonia* and *J. rubens* extracts in CCL_4_/mice. Pre-treatment and post-treatment of *P. pavonia* and *J. rubens* extracts in CCL_4_/mice increased WBCs count and lymphocytes relative numbers and reduced the neutrophils and monocytes relative numbers. Pre-treatment and post-treatment of _CCL4_/mice with *P. pavonia* and *J. rubens* extracts significantly reduced the release amounts of pro-inflammatory cytokines TNF-α and IL-6 and significantly inhibited the increased CRP level. Furthermore, pre-treatment and post-treatment of CCL_4_/mice with *P. pavonia* and *J. rubens* extracts recovered the activities of GSH, and significantly decreased MDA level. CCL4/mice pre-treated and post-treated with *P. pavonia* and *J. rubens* extracts decreased alanine aminotransferase (ALT) and aspartate aminotransferase (AST) levels. Pre- and post-treatment of CCL_4_/mice with the *P. pavonia* and *J. rubens* extracts ameliorated the liver damages caused by CCl_4_ and significantly inhibited the necrotic area, indicating hepatic cell death and decreased periportal hepatic degeneration, fibrosis, and inflammation.

## Introduction

Hepatocellular impairment and inflammatory cell infiltration result in cirrhosis and fibrosis^1^. One of the inflammatory mediators is the tumor necrosis factor-alpha (TNF-α), interleukin 6 (IL-6), and C-reactive protein (CRP) that are secreted primarily by activated natural killer (NK) and T cells and liver, respectively. Pro-inflammatory and anti-inflammatory cytokines are recognized for their immunomodulatory, anti-inflammatory, and antiviral properties^2^. TNF-α and IL-6 contribute to systemic inflammation that enhances the immune acute stage reaction^3^, is associated with chronic inflammation, and enhances tumor formation, growth, and metastasis^4^. IFN-γ-activated macrophages secret plenty of cytokine TNF-α, which is a pleiotropic cytokine secreted when antigen activation that plays crucial roles in the immune response and may also modulate hepatocyte function^2^. Tripathi et al.^5^. reported that TNF aggravates oxidative stress and inflammatory responses- and IL-6 in the hepatocytes. CRP is one of the proteins the liver produces that go up in response to inflammation. Its level increases in response to specific inflammatory cytokines formed by leucocytes during inflammation.

Inflammation is a natural defense mechanism against infection that occurs in reaction to various events, including tissue damage^6,7^. The pathophysiology of many illnesses is caused by immune system malfunction^8,9^. Because of their capacity to recognize, engulf, and eliminate numerous pathogens, phagocytes like macrophages and neutrophils play a critical role in innate immunity^10^. Inflammatory mediators, including TNF-α, IL-6, and CRP, are produced by macrophages and other immune components during the inflammatory response^11,12^. Modulating immune cell-mediated inflammatory responses is critical for developing a novel anti-inflammatory treatment strategy, and immune system modulation is becoming a significant topic in pharmacology^13^. In hepatic inflammation, the damaged hepatocytes and infiltrating inflammatory cells support Kupffer cells, liberating cytokines such as TNF-α, IL-6, and CRP^14^. The Immunomodulation approach in the inflammatory process is related to the nonspecific stimulation of macrophages, monocytes, neutrophils, natural killer (NK) cells, and lymphocytes’ activity and effectiveness, as well as the synthesis of numerous effector immune mediators by activated cells^15^.

The main potentials of carbon tetrachloride (CCl_4_) are liver injury initiation by elevating lipid peroxidation; reduction of antioxidants, such as nonenzymatic reduced glutathione (GSH) and Malondialdehyde (MDA), activities, production of reactive oxygen species (ROS), and increasing of hepatic enzymes aspartate aminotransferase (AST) and alanine aminotransferase (ALT)^16–18^. AST and ALT are sensitive biomarkers in hepatic damage diagnosis. MDA is a lipid peroxidation end product and a marker of reactive oxygen species (ROS). Increased liver inflammation encourages the development of lipid peroxidation products, including MDA^19,20^.

The main effects of brown seaweeds or their compounds are the potential suppression of ROS-initiated inflammatory signaling pathways enhanced by chemokines, toll-like receptors, and nuclear factor kappa-light-chain-enhancer of activated B cells (NF-κB) signaling molecules and immune regulation of innate and adaptive immune responses, particularly by interfering with macrophage and lymphocyte immunity^21^. Consumption of brown seaweed, extracts, or specific chemicals modulates various components of the inflammatory immune response, including steady-state immunological parameters, innate and adaptive immune responses, and inflammation^21^.

The underlying mechanisms of the anti-inflammatory impacts of brown seaweed extracts are linked to different stages of the inflammatory response. Disruption of the steady state of the body tissue results in the immune cells responding to damage or irritation via an innate cascade driving inflammation. Pre-treating macrophages and lymphocytes with the seaweed component fucoidan was reported to suppress the pro-inflammatory reaction or promote anti-inflammatory potentials, leading to NF-kB translocation inhibition and reduced levels of pro-inflammatory mediator synthesis^22,23^.

One of the oxidative stress markers, reactive oxygen species (ROS), is a critical component of the inflammatory response, serving a variety of functions after tissue damage, including acute inflammation initiating, clarifying infection and necrotic tissue, and mediating various intracellular transduction pathways. Seaweed extracts appear to suppress the progression of inflammation, particularly the production of ROS. One of the treatments for inflammation is to reduce the formation of ROS, which gives an interesting aspect for studying the inflammatory immune response of brown seaweed constituents^21^.

Liver illnesses have become one of the most frequent and dangerous diseases worldwide due to changes in human lifestyle. Furthermore, many approaches have investigated new herbal and marine seaweed-derived medications to decrease drug-induced liver damage^24^. As a result of their rich sources of structurally miscellaneous bioactive compounds with immune stimulation, antitumoral, antivirus, immunomodulatory, anti-inflammatory properties, and hepatoprotective properties, marine seaweeds have attracted the attention of pharmaceutical and medical industries^25–30^. Seaweed such as *P. pavonica* and *J. rubens* extracts reduced oval cell proliferation and improved liver architecture, indicating hepatoprotective potentials against chemical-induced liver damage. Previously, researchers have demonstrated that seaweed extracts can reduce vascular inflammation caused by immune cell adhesion^31,32^. The marine algae bioactive gradients may impact the immunological and inflammatory systems, affecting disease development^33,34^. Brown seaweed extracts exhibit relatively high levels of chlorophyll, carotenoids, fucoxanthin polyphenol phlorotannins that have been shown to possess a range of bioactive properties, including anti-oxidant, anti-inflammatory, anti-allergic, anti-cancer, anti-obesity, and neuroprotective activities^35^.

Given these considerations, this paper aimed to investigate the brown and red seaweeds *P. pavonica* and *J. rubens* extracts efficacy on the immune compartments that potentiate consequent responses to inflammatory implications in the carbon tetrachloride (CCl_4_)-induced hepatotoxicity animal models of inflammation by revealing the protective effects of *P. pavonica* and *J. rubens* extracts against chronic hepatic inflammation and possible pathways for its immune stimulation, immunomodulatory, anti-inflammatory, antioxidant and hepatoprotective potentials.

## Materials and methods

### Sample collection

**Plate 1:**
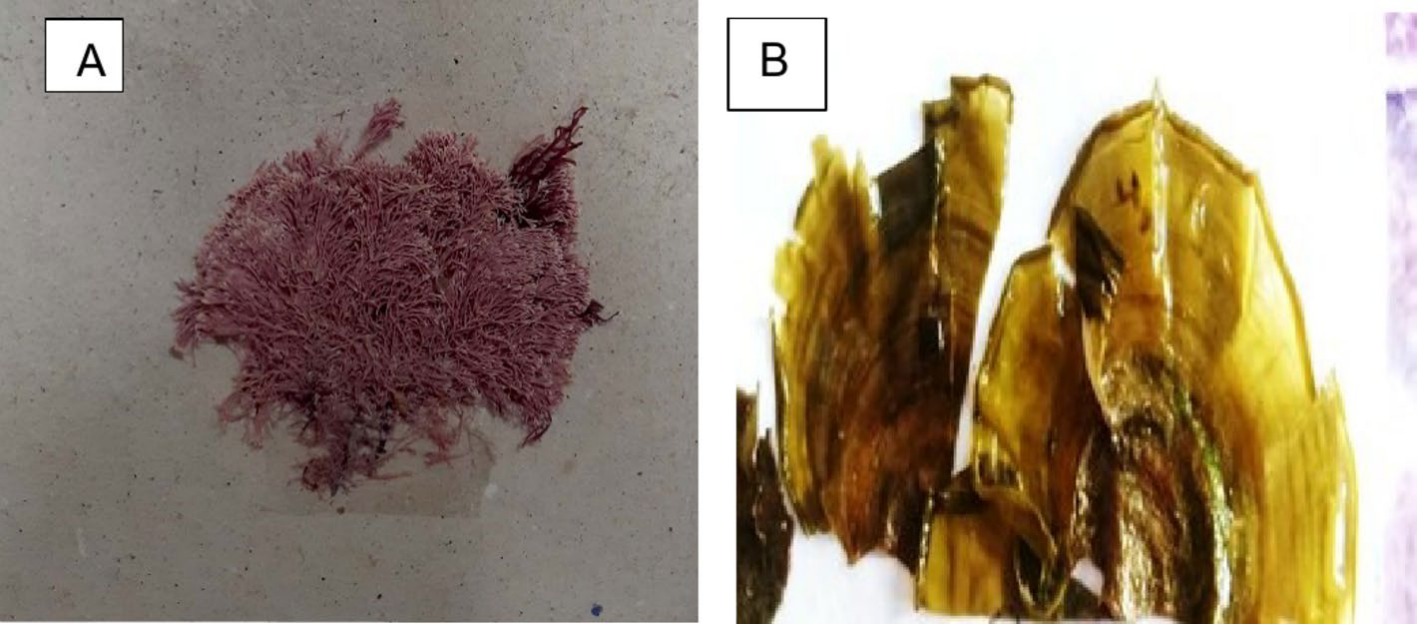
Photo of (**A**) *Jania rubens* and (**B**) *Padina pavonica*.

*P. pavonica* and *J. rubens* (Plate 1) were gathered from Abu Qir Bay, Alexandria, Egypt. Seaweed samples were transported in chilled circumstances to the Faculty of Science, Tanta University, Egypt. After that, collected macroalgae were washed with tap water and then air-dried in the shade at room temperature before being dried at 38 °C in the oven. The dried materials were ground and kept for more studies. The collected seaweeds were identified according to morphological criteria as size, shape, and color in conjunction with reference to marine benthic seaweed literature. The seaweeds were recognized using the Algae Base website to confirm their identification, according to Guiry and Guiry^36^.

### Preparation of crude extracts

*Padina pavonica* and *Jania rubens* extraction process was conducted following El-Sheekh et al.^37^. Briefly, 1 g of seaweed powder was soaked in methanol (1:30 w/v) on a rotary shaker at 120 rpm at room temperature for two days. The supernatant was heated in the oven at 45 °C to eliminate the extra solvent. The crude extracts were dissolved in methanol to a final concentration of 5 mg/mL and kept at −20 °C for future investigations.

### Mice

Seventy female 8-week-old albino mice (25 ± 2 g) were supplied by Theodor Bilharz Research Institute (TBRI), Cairo, Egypt. Animals were distributed into seven groups, ten mice each, and supplied with a standard pellet diet and tap water *ad libitum*. The experimental protocol was conducted according to the ethics of research animals National Institutes of Health (NIH) guidelines and the recommendations of the Institutional Animal Ethical Committee (EAC), Faculty of Science, Tanta University, Tanta, Egypt. This study was approved by the Faculty of Science, Tanta University’s Scientific Induction Ethics Committee, and guidelines for the humane care of animals (IACUC-SCI–TU-0412). It is also confirmed that this study is reported in accordance with ARRIVE guidelines.

### Study design and treatment

Seven groups of mice were used. The 1st, 2nd, and 3rd groups intraperitoneally (i.p.) received saline (normal control), no treatment (CCL_4_ negative control), and drug (silymarin at 50 mg/kg, positive control), respectively. The 4th and 5th groups (post-treatment groups) were intraperitoneally injected with *P. pavonica* extract (2.5 µg/mouse) and *J. rubens* extract (2.3 µg/mouse), respectively, twice a week for seven consecutive weeks post CCL_4_. The 6th and 7th groups (pre-treatment groups), i.p., received *P. pavonica* extract (2.5 µg/mouse) and *Jania. rubens* extract (2.3 µg/mouse), respectively seven days before CCL_4_ injection and then twice a week for seven consecutive weeks post-CCL_4_ injection. Hepatic chronic inflammation in mice groups 2–7 was induced by i.p. administration of 0.5 ml/kg of CCl_4_ (Sigma-Aldrich, USA) suspended in PBS (final volume, 0.1 mL/mouse) twice/week for six constitutive weeks. At the end of the experimentation, mice were sacrificed by cervical dislocation, and sera and blood were collected to calculate the count of white blood cells (WBCs), percentage of lymphocytes, monocytes, and neutrophils, the level of some pro-inflammatory cytokines interleukin 6 (IL-6), tumor necrosis factor-α (TNF-α), inflammatory C-reactive protein (CRP) and antioxidant enzymes glutathione (GSH) and malondialdehyde (MDA) and liver function. Liver tissues were dissected to analyze the histopathological changes in the treated liver. The pre-treatment and post-treatment doses of *P. pavonica* and *J. rubens* extracts were determined according to our previous study^38^.

### Leucocytes analysis

At the end of the experimentation, mice were anesthetized, and blood was drained from their retro-orbital plexus in heparinized microhematocrit tubes. Blood parameters such as white blood cells (WBC) (10^3^/cm) and the relative number of neutrophils, lymphocytes, and monocytes in peripheral blood (PB) were evaluated using a Nihon Kohden automated hematology analyzer (model MEK-6318 K, Japan).

### Sera preparation

The blood of treated mice was collected and left for 3 h to verify complete coagulation. The collected blood was centrifuged for 10 min at 3000 rpm. The sera were collected and held at − 80 °C for biochemical investigations.

### Assay the functions of liver and kidney

Serum aminotransferase enzymes: aspartate aminotransferase (AST) (U/l) and alanine aminotransferase (ALT) (U/l), creatinine (mg/dl), and urea mg/dl) levels were calorimetrically evaluated by a fully automatic biochemical analyzer (Vita lab Selectra E, German) via standard ready-to-use kits (Human Gesellschaft Für Biochemica and Diagnostica MBH, Germany). The manufacturer’s manuals were precisely followed throughout the experiment.

### Assay of malondialdehyde and antioxidants

Liver specimens were rapidly dissected from treated mice. A liver piece was homogenized in ice-cold KCl (1.15% w/v) to get a liver homogenate (10% w/v). Antioxidant enzymes such as malondialdehyde (MDA) and glutathione (GSH) were investigated in liver tissue homogenates by enzyme-linked immunoassay (ELISA) according to the instructor manual of ready-made kits (CUSABIO, USA).

### Assay of inflammatory cytokines and proteins

IL-6, TNF-α, and CRP were estimated by ELISA (enzyme-linked immunoassay) according to the manufacturer’s protocol of ELISA-specific kits (CUSABIO, USA).

### Histopathological examination

The liver tissues were dissected from the mice and fixed in 10% formalin for three days. The fixed liver tissues were dehydrated and rinsed several times in absolute alcohol and then embedded in paraffin. Serial 4–5 μm sections from each group were mounted on glass slides. Slides were stained with hematoxylin and eosin (H&E) dye for microscopic investigation. Under a light microscope, the stained sections were examined and photographed.

### Statistical analysis

Data are expressed as the mean ± standard deviation (*n* = 10). Results were analyzed by one-way analysis of variance (ANOVA), followed by Dunnett’s test, Tukey’s test, or the *t*-test as *post hoc* tests. Probability values less than 0.05 were considered significant.

## Results

### Leucocytes profile

In the present study, i.p. pre-treatment and post-treatment of *P. pavonica* and *J. rubens* extracts remarkably increased WBCs counts in CCL_4_/mice by 2.0 and 2.0-fold for *Padina. pavonia* extract, respectively, and 1.8 and 1.7-fold for *Jania. rubens* extract, respectively, compared to CCL_4_/mice control. Compared to Silymarin-treated CCL_4_/mice (positive control), i.p., pre-treatment and post-treatment of CCL_4_/mice with *Padina. pavonia* increased the total number of WBCs by 1.2 and 1.2-folds, respectively (Table 1). Notably, intraperitoneal pre-treatment and post-treatment of CCL_4_/mice with *P. pavonica* and *J. rubens* extracts successfully increased the total number of WBCs. They revealed a remarkable potential in retaining their total number to be closely related to the naïve mice control (Table 1).

**Table 1:** Cellular alternation in peripheral blood leucocytes profile of CCL_4_/mice treated with PBS, silymarin, *P. Pavonia* and *J. Ruben extracts*.

Treatments		WBC (× 10^3^)	Leucocytes differentials relative number (%)		
			Lymphocytes	Neutrophils	Monocytes
Control		9.10 ± 1.70	82.70 ± 4.40	12.10 ± 6.70	6.90 ± 0.78
CCL_4_		4.70 ± 0.40	70.90 ± 7.00	16.00 ± 5.90	7.90 ± 0.11
CCL_4_ + Silymarin		8.10 ± 1.60	75.80 ± 7.50	13.20 ± 5.10	6.90 ± 0.65
*P. pavonia*	Therapeutic dose	9.60 ± 5.70	61.60 ± 5.60	25.30 ± 6.40	7.80 ± 0.06
	Prophylactic dose	9.80 ± 5.90	72.50 ± 13.80	12.10 ± 6.20	7.40 ± 0.92
*J. rubens*	Therapeutic dose	8.60 ± 3.50	71.00 ± 11.50	13.80 ± 6.70	7.20 ± 0.75
	Prophylactic dose	8.40 ± 1.90	76.10 ± 8.20	14.90 ± 4.90	5.30 ± 2.30

Data were represented as mean ± SD.

I.p. pretreatment of CCL_4_/mice with *Padina pavonia* extract and i.p. pre-treatment and post-treatment of CCL_4_/mice with *Jania rubens* extract increased the lymphocytes relative number compared to CCL_4_/mice control. In addition, CCL_4_/mice pre-treated with *Jania rubens* extract recorded higher lymphocytes relative number compared with CCL_4_/mice control and Silymarin-treated CCL_4_/mice (positive control) (Table 1).

Noticeably, i.p. post-treatment of CCL_4_/mice with *Padina pavonia* extract remarkably increased the neutrophil relative number by 2.0, 1.6, and 1.9-fold compared to naïve mice control, naïve CCL_4_/mice control, and Silymarin-treated CCL_4_/mice (positive control), respectively (Table 1). Furthermore, i.p. pre-treatment and post-treatment of CCL_4_/mice with *J. rubens* extract increased the neutrophil relative number by 1.2- and 1.3-fold compared to naïve mice control. Additionally, CCL_4_/mice pre-treated with *J. rubens* recorded an increased relative number of neutrophils by 1.2-fold compared to Silymarin-treated CCL_4_/mice (positive control) (Table 1).

I.p. pre-treatment and post-treatment of CCL_4_/mice with *P. pavonica* and *J. rubens extracts* resulted in a noticeable nonsignificant reduction of monocytes relative number that reached the least value in CCl_4_/mice pre-treated with *J. rubens* extract compared to naïve CCL_4_/mice control (Table 1). Furthermore, CCL_4_/mice pre-treated and post-treated with *P. pavonica* extracts recorded an increase in the relative number of monocytes by 1.2 and 1.3, respectively, and their post-treatment with *Jania rubens* extract increased the relative number of monocytes by 1.1-fold compared to Silymarin-treated CCL_4_/mice (positive control) (Table 1).

### Assay of inflammatory cytokines and proteins

**Fig. 1:**
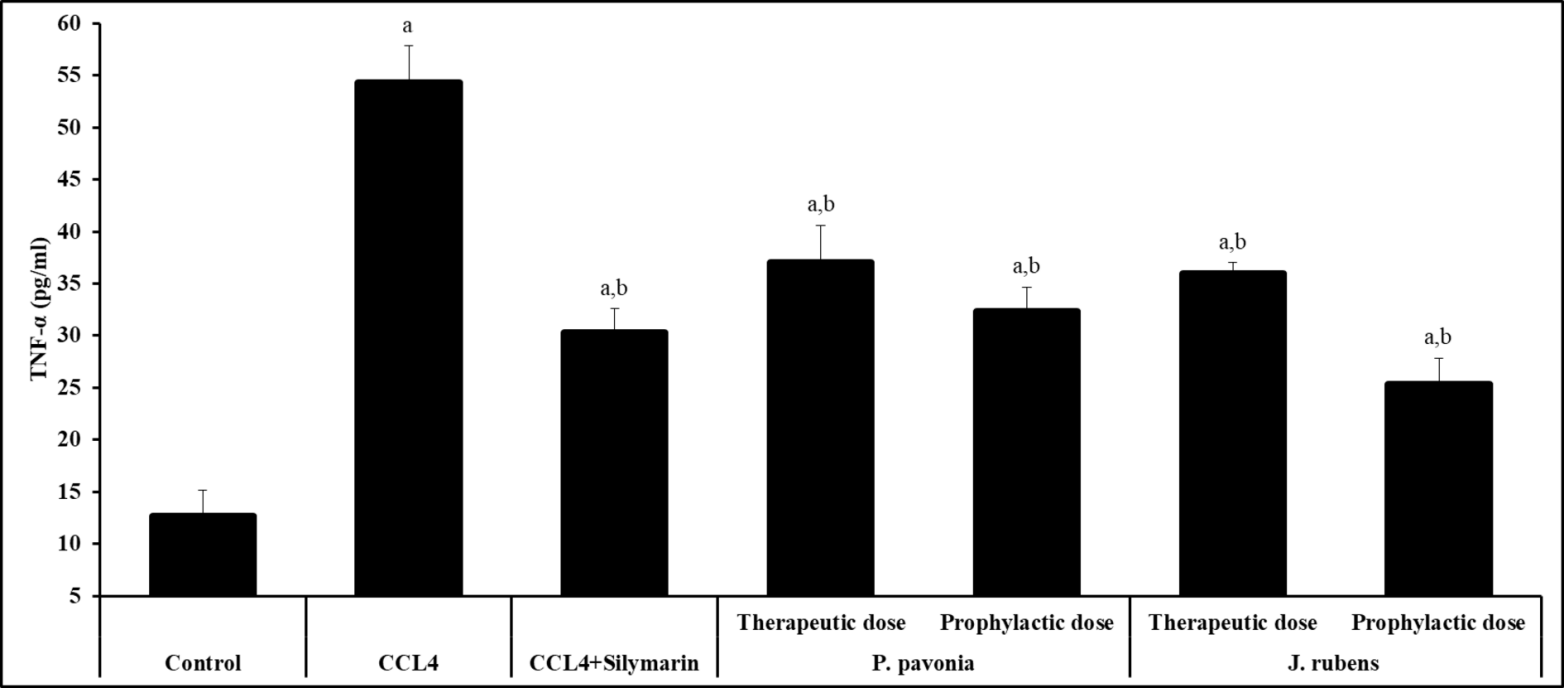
Inhibitory effects of *P. pavonia* and *J. rubens* extracts on TNF-α (pg/ml) in CCl_4_-induced liver injury mice. Data were represented as mean ± SD. The difference between groups was considered statistically significant at *P* < 0.05. CCL_4_/mice (*n* = 10) were i.p inoculated with *P. pavonia* extract (therapeutic dose: 2.5 µg/mouse; prophylactic dose: 2.5 µg/mouse), *J. rubens* (therapeutic dose: 2.3 µg/mouse; prophylactic dose: 2.3 µg/mouse), Silymarin (50 mg/kg) or phosphate buffer saline (PBS). ^a^Statistically significant vs. mice received PBS alone (normal control). ^b^Statistically significant vs. CCL_4_/mice control group received PBS alone (negative control).

As shown in Fig. 1, CCL_4_/mice pre- and post-treated with *P. pavonica* and *Jania rubens* extracts significantly reduced serum TNF-α levels compared to naïve CCL_4_/mice control. Additionally, the pre-treatment of CCL_4_/mice with *J. rubens* extract led to a nonsignificant decrease in serum TNF-α concentration compared to silymarin-treated CCL_4_/mice (positive control) (Fig. 1). Noticeably, CCL_4_/mice pre-treated with *P. pavonica* and *J. rubens* extracts recorded the best results in significantly decreasing the serum TNF-α concentration compared to the naïve CCL_4_/mice control and silymarin-treated CCL_4_/mice (positive control) (Fig. 1).

**Fig. 2:**
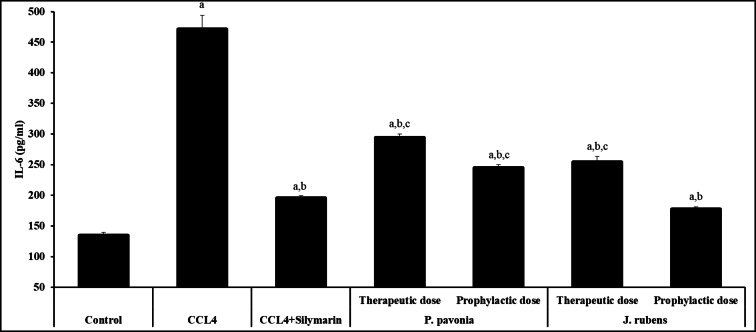
Inhibitory effects of *P. pavonia* and *J. rubens* extracts on IL-6 (pg/ml) in CCl_4_-induced liver injury mice. Data were represented as mean ± SD. The difference between groups was considered statistically significant at *P* < 0.05. CCL_4_/mice (*n* = 10) were i.p inoculated with *P. pavonia* extract (therapeutic dose: 2.5 µg/mouse; prophylactic dose: 2.5 µg/mouse), *J. rubens* (therapeutic dose: 2.3 µg/mouse; prophylactic dose: 2.3 µg/mouse), Silymarin (50 mg/kg) or phosphate buffer saline (PBS). ^a^Statistically significant vs. mice received PBS alone (normal control). ^b^Statistically significant vs. CCL_4_/mice control group received PBS alone (negative control). ^c^Statistically significant vs. CCL_4_/mice group received Silymarin (positive control).

The effectiveness of the *P. pavonica* and *J. rubens* extracts on the serum IL-6 concentration has been investigated as shown in Fig. 2. I.P. pre-treatment and post-treatment of CCL_4_/mice with *P. pavonica* and *J. rubens* extracts resulted in a significant reduction in serum IL-6 level compared to naïve CCL_4_/mice control. CCL_4_/mice i.p. pre-treated and post-treated with *P. pavonica* and post-treated with *J. rubens* recorded a significant increase in the level of serum IL-6 compared to silymarin-treated CCL_4_/mice (positive control). Contrarily, i.p. pre-treatment of CCL_4_/mice with *J. rubens* extract showed a significant decrease in serum IL-6 concentration compared to Silymarin-treated CCL_4_/mice (positive control) (Fig. 2).

**Fig. 3:**
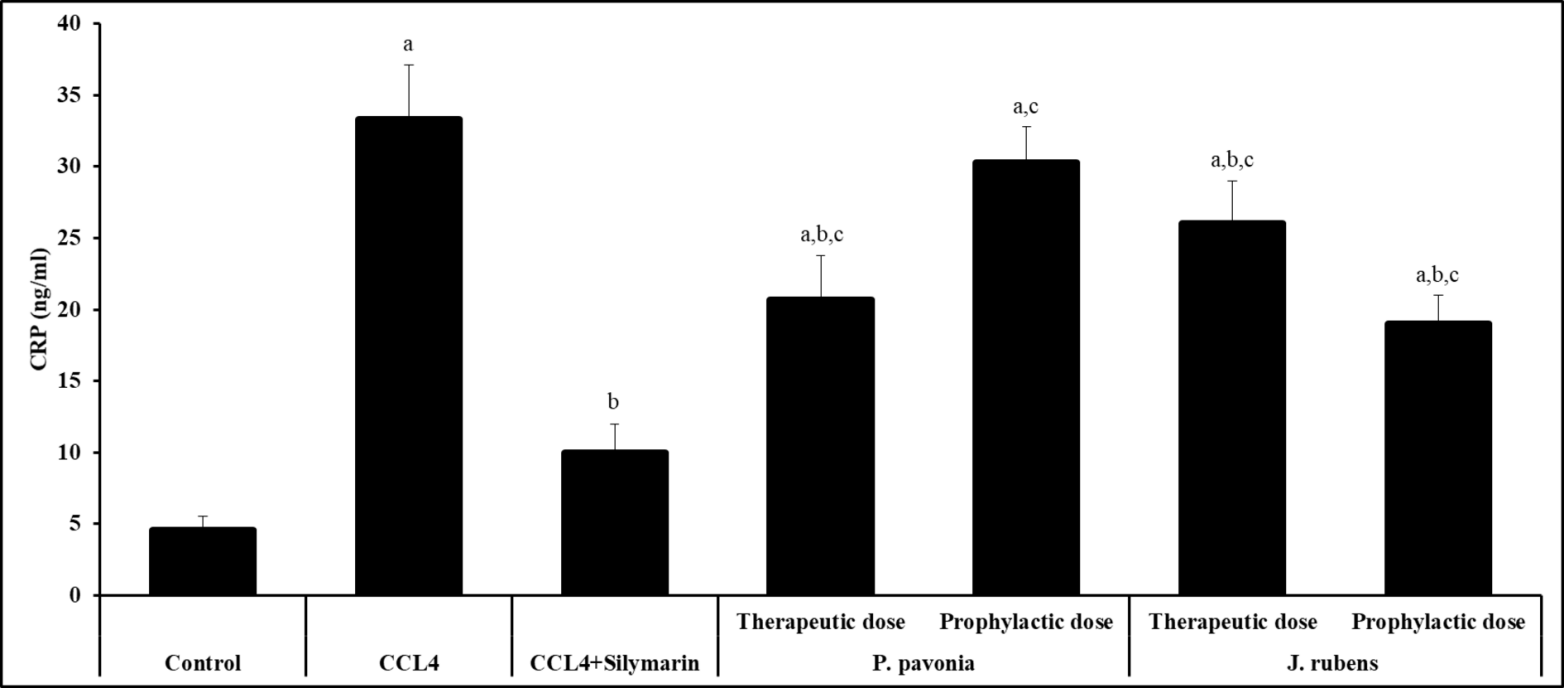
Inhibitory effects of *P. pavonia* and *J. rubens* extracts on CRP (ng/ml) in CCl_4_-induced liver injury mice. Data were represented as mean ± SD. The difference between groups was considered statistically significant at *P* < 0.05. CCL_4_/mice (*n* = 10) were i.p inoculated with *P. pavonia* extract (therapeutic dose: 2.5 µg/mouse; prophylactic dose: 2.5 µg/mouse), *J. rubens* (therapeutic dose: 2.3 µg/mouse; prophylactic dose: 2.3 µg/mouse), Silymarin (50 mg/kg) or phosphate buffer saline (PBS). ^a^Statistically significant vs. mice received PBS alone (normal control). ^b^Statistically significant vs. CCL_4_/mice control group received PBS alone (negative control). ^c^Statistically significant vs. CCL_4_/mice group received Silymarin (positive control).

Interestingly, i.p., pre-treatment and post-treatment of CCL_4_/mice with the extracts of *P. pavonica* and *J. rubens* significantly decreased the serum level of CRP compared to naïve CCL_4_/mice control. Additionally, pre-treatment and post-treatment of CCL_4_/mice *P. pavonica* and *J. rubens* extracts produced the highest significant inhibition in the level of serum CRP comparing to the CCL_4_ control group and silymarin-treated CCL_4_/mice (positive control) (Fig. 3). Intraperitoneal pre-treatment and post-treatment of CCL_4_/mice with *P. pavonica and J. rubens* significantly increased the serum level of CRP compared to silymarin-treated CCL_4_/mice (positive control) (Fig. 3).

### Assay of malondialdehyde and antioxidants

**Fig. 4:**
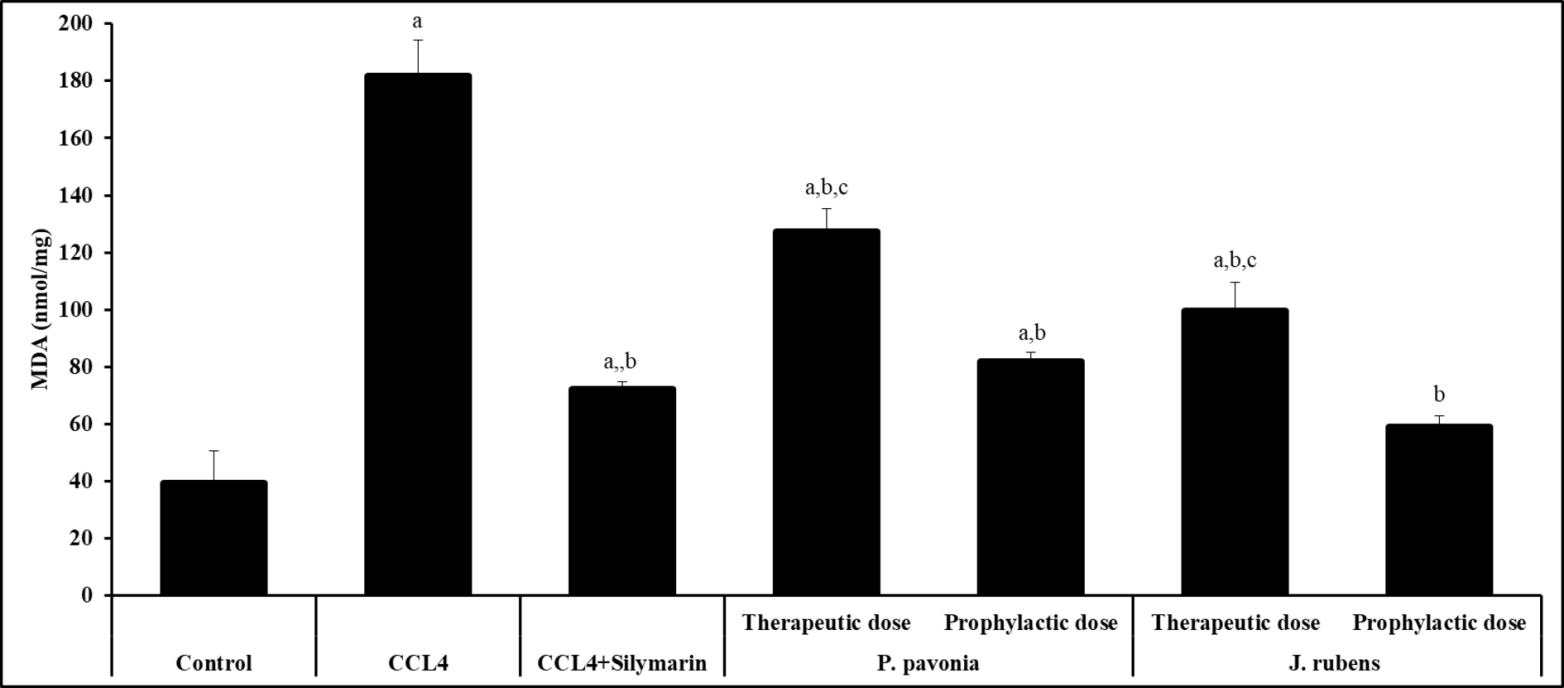
Inhibitory effects of *P. pavonia* and *J. rubens* extracts on MDA (nmol/mg) in CCl_4_-induced liver injury mice. Data were represented as mean ± SD. The difference between groups was considered statistically significant at *P* < 0.05. CCL_4_/mice (*n* = 10) were i.p inoculated with *p. pavonia* extract (therapeutic dose: 2.5 µg/mouse; prophylactic dose: 2.5 µg/mouse), *J. rubens* (therapeutic dose: 2.3 µg/mouse; prophylactic dose: 2.3 µg/mouse), Silymarin (50 mg/kg) or phosphate buffer saline (PBS). ^a^Statistically significant vs. mice received PBS alone (normal control). ^b^Statistically significant vs. CCL_4_/mice control group received PBS alone (negative control). ^c^Statistically significant vs. CCL_4_/mice group received Silymarin (positive control).

**Fig. 5:**
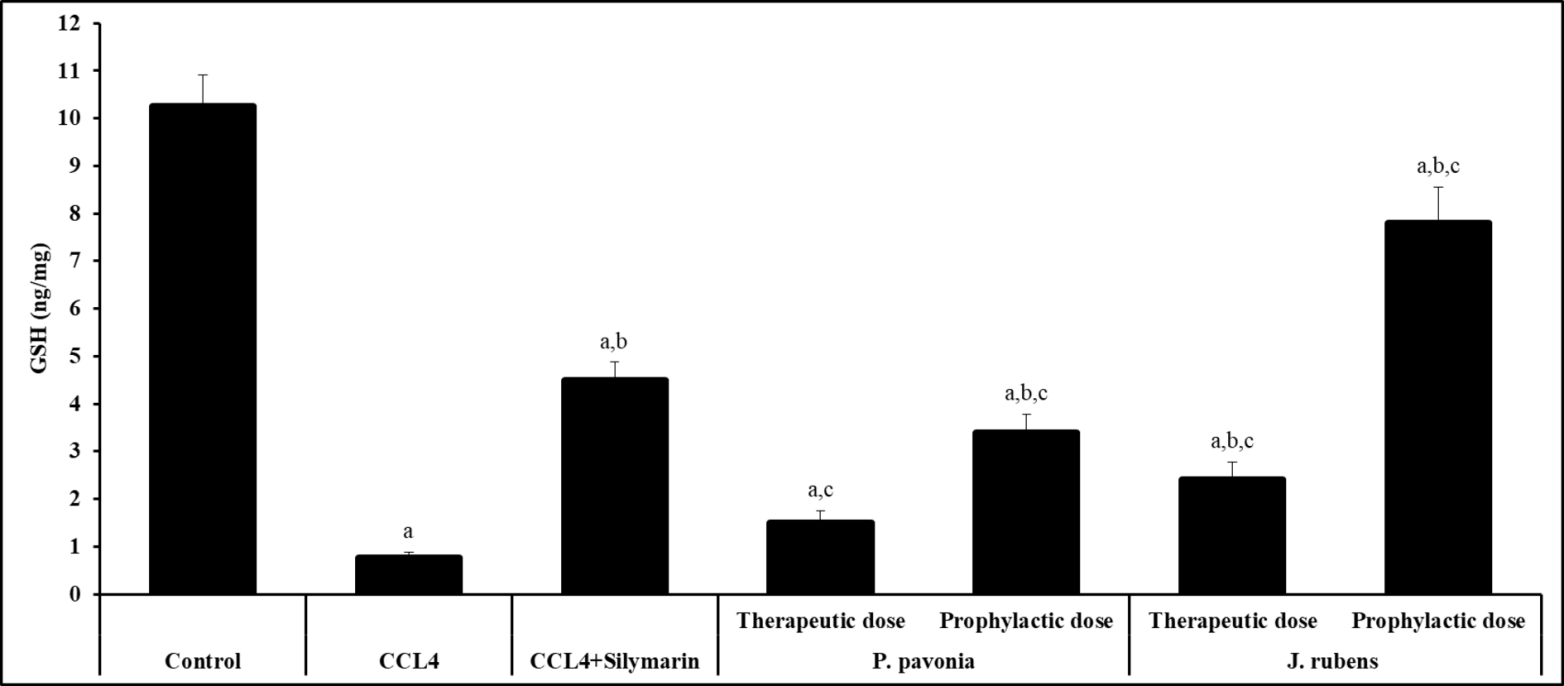
Attenuated effects of *P. pavonia* and *J. rubens* extracts on GSH (ng/mg) in CCl_4_-induced liver injury mice. Data were represented as mean ± SD. The difference between groups was considered statistically significant at *P* < 0.05. CCL_4_/mice (*n* = 10) were i.p inoculated with *P. pavonia* extract (therapeutic dose: 2.5 µg/mouse; prophylactic dose: 2.5 µg/mouse), *J. rubens* (therapeutic dose: 2.3 µg/mouse; prophylactic dose: 2.3 µg/mouse), Silymarin (50 mg/kg ) or phosphate buffer saline (PBS). ^a^Statistically significant vs. mice received PBS alone (normal control). ^b^Statistically significant vs. CCL_4_/mice control group received PBS alone (negative control). ^c^Statistically significant vs. CCL_4_/mice group received Silymarin (positive control).

Oxidative stress plays a key role in liver damage. Hepatotoxicity results in rising ROS generation in hepatocytes, triggering GSH depletion and MDA, lipid peroxidation end product, and elevation. MDA is an indicator of oxidative stress. ROS production also caused a significant reduction in the activity of antioxidant enzymes, such as GSH. Oxidative stress parameters were evaluated by measuring the levels of GSH and MDA due to understand the possible mechanisms of the observed hepatoprotective effects. The administration of mice with CCl_4_ resulted in severe liver injury, as showed by the marked elevation in MDA level (Fig. 4). As can be seen in Fig. 1, MDA contents were significantly decreased in CCL_4_/mice pre-treated and post-treated with *P. pavonica* and *J. rubens* extract compared to those in the naïve CCL_4_/mice control. The data showed that i.p., pre-treatment of CCL_4_/mice with *P. pavonica* and *J. rubens* extracts produced the highest significant decrease in the hepatic MDA level compared to the naïve CCL_4_/mice control. Meanwhile, hepatic MDA levels decreased significantly in CCL_4_/mice pre-treated with *Jania rubens* extract compared to silymarin-treated CCL_4_/mice (positive control) (Fig. 4). However, hepatic GSH level increased significantly in CCL_4_/mice post-treated with *P. pavonica* and *J. rubens* extracts compared to naïve CCL_4_/mice control. Post-treatment of CCL_4_/mice with *Padina pavonia* and *Jania rubens* extract led to a significant increase in hepatic GSH level and their pre-treatment with *J. rubens* extract resulted in a significant increase in hepatic GSH level compared to Silymarin-treated CCL_4_/mice (positive control) (Fig. 5). However, the i.p. pre-treatment and post-treatment of CCL_4_/mice with *P. pavonica* and *J. rubens* decreased the MDA level in liver tissues (Fig. 4), and the diminished GSH levels were retained (Fig. 5). These results suggest that *P. pavonica* and *J. rubens* extract exerts hepatoprotective effects by alleviating oxidative.

### Assay of liver and kidney function

Post-treatment of CCL_4_/mice with *P. pavonica* extract significantly decreased the level of serum AST. Their pre-treatment with *P. pavonica* nonsignificantly reduced the level of serum AST compared to CCL_4_/mice control. Contrarily, i.p. post-treatment of CCL_4_/mice with *J. rubens* extract significantly increased the level of serum AST and their pre-treatment with *J. rubens* extract nonsignificant increased the level of serum AST compared to naïve CCL_4_/mice control (Table 2). Additionally, i.p. post-treatment of CCL_4_/mice with *P. pavonica* extract nonsignificant decreased the level of serum AST compared to Silymarin-treated CCL_4_/mice. Meanwhile, CCL_4_/mice post-treated with *J. rubens* extract recorded a significant increase in the level of serum AST, and their pre-treatment with *J. rubens* nonsignificant increased the level of serum AST compared to Silymarin-treated CCL_4_/mice (positive control) (Table 2). Moreover, i.p. pre-treatment and post-treatment of CCL_4_/mice with *P. pavonica* and *J. rubens* extract significantly decreased the level of serum ALT compared to naïve CCL_4_/mice control. Additionally, i.p. post-treatment of CCL_4_/mice with *P. pavonica* extract nonsignificant decreased the level of serum ALT that was significantly increased in CCL_4_/mice pre-and post-treated with *J. rubens* extract compared to Silymarin-treated CCL_4_/mice (positive control) (Table 2).

**Table 2:** The inhibitory effects of *P. Pavonia* and *J. Rubens* extracts on the level of hepatic marker enzymes; serum aspartate aminotransferase (AST) (U/l), alanine aminotransferase (ALT) (U/l) in CCl_4_-induced liver injury mice.

Treatments		Liver marker enzymes	
		AST (U/l)	ALT (U/l)
Control		127.00 ± 9.90	83.90 ± 12.20
CCL_4_		199.00 ± 15.70^a^	264.10 ± 11.10^a^
CCL_4_ + Silymarin		183.70 ± 9.30^a^	130.70 ± 12.30^a, b^
*P. pavonia*	Therapeutic dose	146.90 ± 10.90^b^	162.20 ± 14.80^a, b^
	Prophylactic dose	184.20 ± 17.40^a^	95.60 ± 21.50^b^
*J. rubens*	Therapeutic dose	282.10 ± 27.90^a, b,c^	224.70 ± 10.90^a, b,c^
	Prophylactic dose	223.40 ± 26.70^a^	193.50 ± 12.80^a, b,c^

Data were represented as mean ± SD. The difference between groups was considered statistically significant at *P* < 0.05. CCL_4_/mice (*n* = 10) were i.p inoculated with *p. pavonia* extract (therapeutic dose: 2.5 µg/mouse; prophylactic dose: 2.5 µg/mouse), *J. rubens* (therapeutic dose: 2.3 µg/mouse; prophylactic dose: 2.3 µg/mouse), Silymarin (50 mg/kg) or phosphate buffer saline (PBS).

^a^Statistically significant vs. mice received PBS alone (normal control).

^b^Statistically significant vs. CCL_4_/mice control group received PBS alone (negative control).

^c^Statistically significant vs. CCL_4_/mice group received Silymarin (positive control).

Intraperitoneal pre- and post-treatment of CCL_4_/mice with *P. pavonica* and *J. rubens* extract significantly decreased serum urea levels compared to naïve CCL_4_/mice control (Table 3). Intraperitoneal pre- and post-treatment of CCL_4_/mice with *P. pavonica* extract markedly decreased serum urea levels compared to Silymarin-treated CCL_4_/mice (positive control) (Table 3). Meanwhile, CCL_4_/mice pre- and post-treated with *P. pavonica* extract recorded a marked decrease in serum creatinine level compared to Silymarin-treated CCL_4_/mice (positive control) (Table 3).

**Table 3:** The inhibitory effects of *P. Pavonia* and *J. Rubens* extracts on the level of kidney biochemical markers; creatinine (mg/dl) and urea (mg/dl) in CCl_4_-induced liver injury mice.

Treatments		Kidney biochemical markers	
		Creatinine (mg/dl)	Urea (mg/dl)
Control		0.64 ± 0.05	37.70 ± 2.50
CCL_4_		0.97 ± 0.05^a^	94.70 ± 7.02^a^
CCL_4_ + Silymarin		0.91 ± 0.04^a^	69.00 ± 5.60^a, b^
*P. pavonia*	Therapeutic dose	0.68 ± 0.16^b, c^	60.00 ± 4.00^a, b^
	Prophylactic dose	0.77 ± 0.03	67.70 ± 2.50^a, b^
*J. rubens*	Therapeutic dose	1.06 ± 0.07^a^	89.00 ± 4.60^a, b,c^
	Prophylactic dose	0.99 ± 0.05^a^	76.30 ± 3.50^a, b^

Data were represented as mean ± SD. The difference between groups was considered statistically significant at *P* < 0.05. CCL_4_/mice (*n* = 10) were i.p inoculated with *P. pavonia* extract (therapeutic dose: 2.5 µg/mouse; prophylactic dose: 2.5 µg/mouse), *J. rubens* (therapeutic dose: 2.3 µg/mouse; prophylactic dose: 2.3 µg/mouse), Silymarin (50 mg/kg) or phosphate buffer saline (PBS).

^a^Statistically significant vs. mice received PBS alone (normal control).

^b^Statistically significant vs. CCL_4_/mice control group received PBS alone (negative control).

^c^Statistically significant vs. CCL_4_/mice group received Silymarin (positive control).

### Histopathological examination

**Fig. 6:**
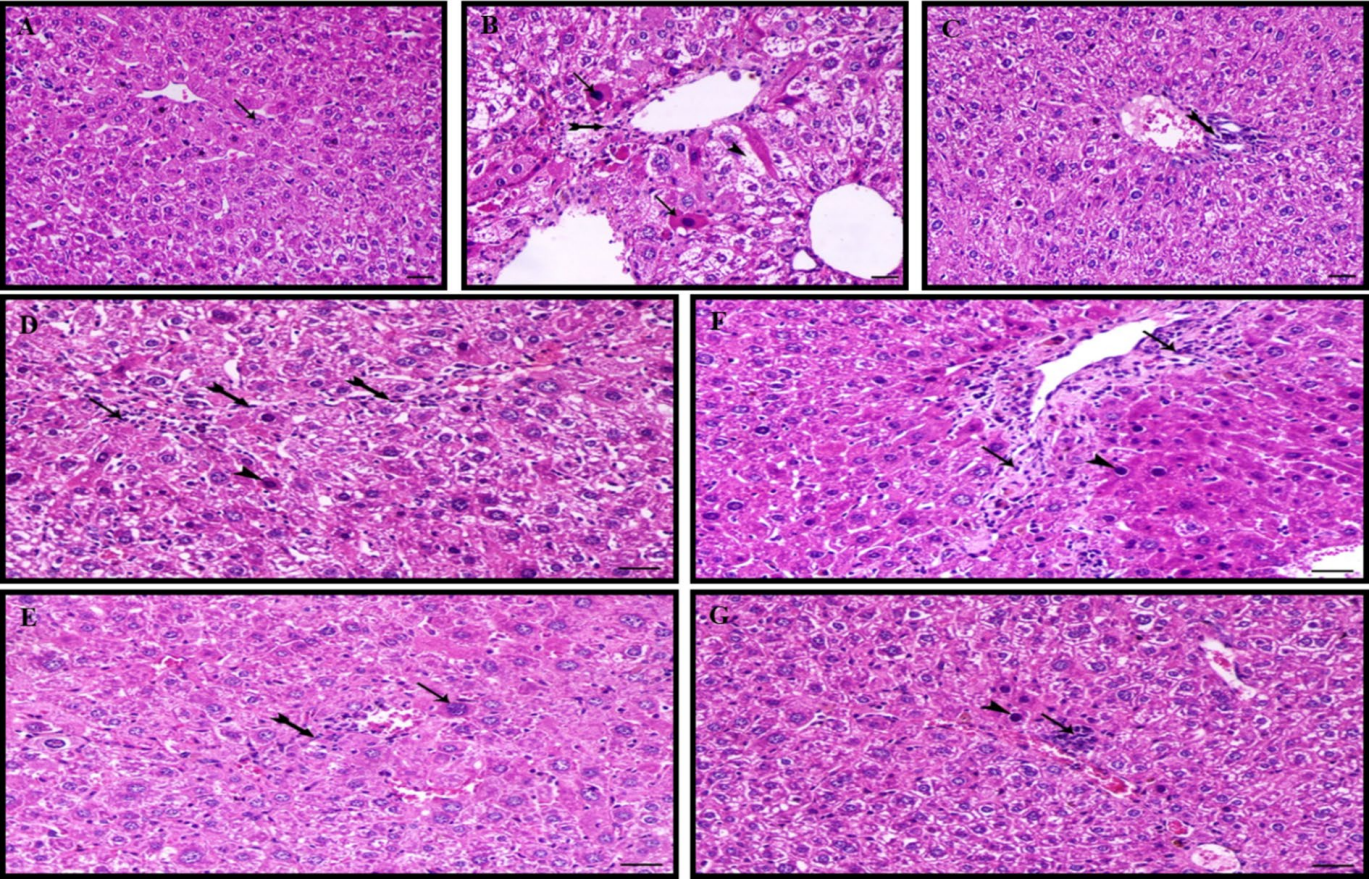
Histopathological graphs of liver sections stained by hematoxylin and eosin (H&E). (**A**) Naïve mice received PBS alone (Normal control), showing normal hepatocytes arranged in cords around the central vein with oval cytoplasm and vesicular-shaped nucleus. (**B**) CCL_4_/mice received PBS (negative control) showing hepatic degenerative and necrotic changes (arrow), cytoplasmic eosinophilia and nuclear pyknosis (arrowhead), hepatocytes vacuolation with perivascular fibroblastic activity and periportal and perilobular hepatic fibrosis (tailed-arrow). (**C**) CCL_4_/mice received Silymarin (50 mg/kg) (positive control) showing moderate degree of periportal hepatic fibrosis (tailed-arrows). (**D**) CCL_4_/mice received therapeutic dose of *P. pavonia* extract (2.5 µg/mouse) showing mild to moderate degree of fibroblastic cells activity (tailed-arrow) and decrease both hepatic degeneration (arrow head) and inflammation (arrow). (**E**) CCL_4_/mice received prophylactic dose of *P. pavonia* (2.5 µg/mouse) showing prominent hepatic regeneration (arrow) and marked decrease of periportal hepatic fibrosis (tailed-arrow). (**F**) CCL_4_/mice received therapeutic dose of *J. rubens* (2.3 µg/mouse) showing hepatic fibrosis limited to the periportal area and collagen deposition (arrow), portal inflammation, mononuclear inflammatory cells infiltration (arrow), indicates smudge-shaped pyknotic nucleus (arrowhead). (**G**) CCL_4_/mice received prophylactic dose of *J. rubens* (2.3 µg/mouse) showing marked decrease of inflammation (arrow) and decrease of periportal hepatic degeneration (arrowhead). X200, bar = 40 μm.

Light microscopic examination of the liver sections of the normal control group received PBS alone revealed normal hepatocytes organized in cords around the central vein (Fig. 6A). Still, the CCL_4_ control group recorded marked hepatic degenerative changes indicating cytoplasmic eosinophilia and nuclear pyknosis and vacuolation of hepatocytes associated with perivascular fibroblastic activity marked hepatic necrotic changes associated with obvious periportal and per lobular hepatic fibrosis (Fig. 6B). Liver sections of the CCL_4_/mice that received Silymarin (positive control) detected mild to moderate periportal hepatic fibrosis (Fig. 6C). On the other hand, i.p. pre- and post-treatment of CCL_4_/mice with *J. rubens* extracts detected a marked decrease in periportal hepatic degeneration and inflammation. In contrast, i.p., pre-and post-treatment of CCL_4_/mice with *P. pavonica* extracts showed a marked decrease in periportal hepatic fibrosis and prominent hepatic regeneration (Fig. 6D–G). It implies that *P. pavonica* and *Jaina rubens* extracts may have hepatoprotective effects against liver damage (Fig. 6D-G). Liver sections of CCL_4_/mice post-treated with *J. rubens* extract showed hepatic fibrosis limited to the periportal area (collagen deposition) and portal inflammation (mononuclear inflammatory cells infiltration), smudge-shaped pyknosis nucleus (Fig. 6F).

Furthermore, liver sections of diseased animals post-treated with *P. pavonica* extract showed mild to moderate fibroblastic cell activity and decreased hepatic degeneration and inflammation (Fig. 6D). In general, pre-treatment of CCL_4_/mice with *P. pavonica* and *J. rubens* extracts showed superior ameliorative histological results of liver sections equally or nearly to the normal control group received PBS alone and this indicated the ability of methanol extract of *Jania rubens* and *P. pavonica* to keep hepatoprotective potentials against liver CCl_4_ poisoning (Fig. 6A–G). The histopathological impairments in the hepatic tissue in all mice groups are briefly shown in Table 4.

**Table 4:** Scores of different hepatic lesions of CCL_4_/ mice treated with silymarin, *P. Pavonia* and *J. Ruben* extracts.

Treatments		Vascular lesions	Degeneration and necrosis	Inflammation	Fibrosis	Extent of lesions
Control		–	–	–	–	–
CCL_4_		++++	++++	+++	+++	+++
CCL_4_ + Silymarin		+	+	++	++	++
*P. pavonia*	Therapeutic dose	++	++	++	++	++
	Prophylactic dose	+	+	+	+	+
*J. rubens*	Therapeutic dose	++	++	++	++	++
	Prophylactic dose	+	+	+	+	+

(–) means no detectable lesions; (+) indicates mild lesions; (++) indicates moderate lesions; (+++) indicates severe focal lesions; (++++) indicates severe diffuse lesions.

## Discussion

Chronic inflammation damages hepatocytes over time, resulting in hepatic inflammation and fibrogenesis caused by a range of injuries in the liver, indicating common pathogenesis^39^. Inflammation and fibrogenesis are accompanied by the recruitment and the migration of the immune components such as lymphocytes, neutrophils, macrophages, monocytes, Kupffer cells, and the inflammatory and proinflammatory mediators TNF-α, IL-6, and CRP that are important in the hepatic inflammation and fibrosis progression and immune defense mechanism against liver injury^40,41^. The need for natural therapies for inflammatory treatment and prevention is attaining rising popularity in contemporary therapeutics. Marine seaweed compounds may influence the immune and inflammatory systems, affecting hepatic inflammation and the progression of fibrogenesis. The potential effects of *P. pavonia* and *Jania rubens* extracts against chronic liver inflammation and a possible mechanistic pathway for its hepatoprotective, immune stimulation, immunomodulatory, antioxidant, and anti-inflammatory activity were investigated in the present study.

### Leucocytes profile

In the current study, i.p., pre- and post-treatment of CCL_4_/mice with *P. pavonica* or *J. rubens* extracts significantly increased WBCs count and lymphocytes relative number compared to the naïve CCL_4_/mice that recorded a significant decrease in WBCs count that could be due to the immune system’s defense function and resulted in a significant decrease in blood lymphocyte population that was described by the lowering in immune activity by long-term liver injury in CCL_4_ intoxication^42,43^. These results conformed with the data of Lavakumar et al.^44^. , who reported a reduction in WBCs profile, including lymphocytes in tumor-induced mice, which was recovered and associated with an increase in WBCs counts and lymphocytes relatives number in the mice treated with seaweeds *Acanthophora spicifera* extract. CCL_4_ metabolism induces ROS that triggered a significant reduction in leukocyte count (leucopoenia) in peripheral blood of mice^45,46^. The potential of ROS, an inflammatory-producing candidate in hepatic intoxication, to enhance WBCs count suggests that they can impact defensive mechanisms and mediate inflammation and fibrogenesis^47^. Our results indicated that the neutrophils and monocytes relative numbers become pretty close to that in naïve mice and Silymarin-treated CCL_4_/mice in CCL_4_/mice groups pre- and post-treated with *Padina pavonia* and *Jania rubens* extract. The abnormal hematologic parameter alternations in total and differential leukocytes induced by hepatic inflammatory response result in the development of many cells from bone marrow, involving neutrophils, which subsequently produce H_2_O_2_ that might destroy surrounding tissues and cells^48^. Monocytes can extravasate into inflammatory sites, distinguish into dendritic cells and tissue macrophages, and respond to infections^49^. In pathological conditions, monocytes can react to various stimuli, reducing inflammation^50^. This investigation revealed that *P. pavonica* and *Jania rubens* extracts can preserve the hematopoietic cells from the damaging effects of hepatic toxicity, and this protection might be due to their antioxidative mechanism and increasing the immune cell types involved in the primary defense mechanism. It is well-known that liver macrophages, particularly activated Kupffer cells, are well known for their roles in initiating, maintaining, and even limiting inflammation in hepatocytes^51,52^. Based on our in vivo findings, *P. pavonica* and *J. rubens* extract is thought to target liver macrophages to suppress uncontrolled inflammatory responses, inhibiting further hepatic damage and enhancing liver tissue repairing. However, this hypothesis needs more studies.

### Assay of inflammatory cytokines and proteins

Our data revealed that along with the increased number of WBCs, the serum levels of TNF-α, IL-6, and CRP in CCL_4_/mice pre- and post-treated with t *Padina pavonia* and *Jania rubens* extract CCL_4_-treated mice significantly decreased compared to the CCL_4_/mice group, which significantly increased. The current study revealed that the treatment of CCL_4_/mice with *P. pavonica* and *J. rubens* extracts substantially reduced CCL_4_-induced liver inflammatory damage by inhibiting TNF-, IL-6, and CRP and by enhancing immune defense mechanism by recruitment and migration of the immune components such as lymphocytes, neutrophils, and monocytes that are the critical candidate in the liver inflammation progression and immune defense mechanism against liver damage^39^. Although CCL_4_ metabolism is linked to liver injuries, secondary damage occurs due to the inflammatory progression triggered by Kupffer cell stimulation^53^. Kupffer cells stimulate the production of inflammatory mediators TNF-, IL-6, and CRP, which are potent inflammatory cytokines implicated in macrophage activation, neutrophil infiltration, and other aspects of inflammation that worsen both inflammatory responses and oxidative stress^5,54–57^. NF-κB is a transcription factor known to be stimulated by ROS. Activated NF-κB enhances the production of several inflammatory mediators, including TNF-α, IL-6, and CRP^58^. Here, CCl_4_ augmented the secretion of liver NF-κB and consequently increased serum TNF-α, IL-6, and CRP. Chronic inflammation is well known to precede fibrosis and to be related to cirrhosis development^59^. Some phytochemicals and others of seaweeds extracts have been demonstrated to suppress inflammatory pathways downstream of proinflammatory mediators, inhibiting inflammation^60^. Our findings are in line with the reports of Boshy et *al*^61^. , who revealed that the serum level of TNF-α, IL-6, and CRP decreased in fucoidan extract from seaweeds *Laminaria species*, which may be contributing to its anti-inflammatory activities by suppressing the production of inflammatory cytokines. CRP is a molecule for pattern recognition, attaching to particular molecular configurations typically exposed during cell death or observed on pathogen surfaces. Its fast increment in synthesis within hours after tissue damage or infection indicates that it participates in the defense of the host and is part of the innate immune response^62^. CRP has been shown to have a vital effect on several inflammatory conditions, particularly preserving mice from fatal challenges with bacterial lipopolysaccharides and several inflammation mediators. Certain immunomodulatory cytokines, TNF-α, IL-6, and CRP, can help produce an anti-inflammatory state in the liver and ameliorate the damage severity. Inhibiting the expression of proinflammatory cytokines TNF-α, IL-6, and CRP by the action of seaweeds has significant anti-inflammatory and immunosuppressive characteristics^63^.

The increase of ROS in hepatotoxicity-stressed mice is the main factor in activating the HSCs (Hepatic stellate cells) and producing proinflammatory and inflammatory cytokines and CRP^64^. There is a reciprocal association between oxidative stress and TNF-α, IL-6, and CRP secretion, i.e., oxidative stress raises TNF-α, IL-6, and CRP expression, and TNF-α, IL-6, and CRP enhances oxidative stress^65,66^. Hence, increased TNF-α, IL-6, and CRP expression here may directly result from hepatic CCL_4_ intoxication or an indirect oxidative stress potential. TNF-α, IL-6, and CRP can directly cause intoxication in the hepatic and glomerular cells and likely contribute to the organ damage found in the present work^67,68^. Subsequently, the assessment of the variation in the levels of serum pro-inflammatory cytokines TNF-α and IL-6 and the proinflammatory protein CRP upon *P. pavonica* and *J. rubens* pre- and post-treatment confirmed the involvement of anti-inflammation for *P. pavonia* and *J. rubens* against liver damage, which is consistent with previous findings of Wills and Asha^55^, Tripathi et al.^5^. and Seki et al.^41^. Interestingly, the i.p. therapy of CCl_4_/mice with *P. pavonica* and *J. rubens* extracts significantly decreased and ameliorated TNF-α, IL-6, and CRP levels being very close to the normal levels in the normal control group, which may be attributed to the ability of *P. pavonica* and *J. rubens* extracts to prevent the free radical generation, promote the endogenous antioxidant mechanism beyond their free radical scavenging functions and the anti-inflammatory action^69^.

### Assay of malondialdehyde and antioxidants

Inflammation due to hepatic injury produces excessive ROS in the body, resulting in oxidative stress and lipid peroxidation, leading to decreases in the antioxidant levels and increases in lipid peroxidation marker MDA levels. MDA is a severe factor in hepatic pathogenesis and hepatocyte damage^70–73^. In the present study, a significant increase in the activity of antioxidant enzymes GSH and a significant reduction in the MDA level in the inflammatory diseased liver after pre- and post-treatment of CCL_4_/mice with *P. pavonica* and *J. rubens* extracts indicated the hepatoprotective potentials of several marine seaweeds by scavenging of free radicals produced by chemical-induced hepatic inflammation^74–76^. These findings are in consistent with the findings of previous studies and provide a novel indication of the effects of marine seaweeds extract on oxidative stress and lipid metabolism^70^ providing additional proof that *P. pavonica* and *J. rubens* treatment may enhance the attenuation of chemically induced liver damage. GSH is a vital antioxidant that scavenges ROS and keeps protein sulfhydryl groups.

Additionally, our findings confirmed that *P. pavonica* and *J. rubens* extracts have hepatoprotective impacts against CCl_4_-induced injury through alleviating oxidative stress by recovering the antioxidant enzymes’ activities, restoring the GSH reservoir, and decreasing lipid peroxidation specifically, and alleviating MDA level. Antioxidant enzymes, GSH, scavengers of hepatic ROS, are considered the first defense line against oxidative stress-induced tissue injury by reducing oxidative stress and inhibiting the inflammatory response, which could be mediated by immune defense mechanisms such as immune stimulation and immunomodulation^77^. Previous studies have reported that brown seaweeds components such as *P. pavonica* and *J. rubens* extract could suppress ROS production^78–80^. Knowing that both oxidative stress and inflammatory processes are interlaced, the clinical applications and therapeutic approaches for liver diseases primarily concentrate on antioxidative and anti-inflammatory processes^52,81,82^, we hypothesize that natural products that extracted from brown seaweed such as *P. pavonica* and *J. rubens* could be a promising source to screen effective hepatic disease with high efficiency and low cytotoxicity. Consequently, inhibition of oxidative stress and inflammation plays a significant role in the ameliorative efficacy of seaweeds *P. pavonica* and *J. rubens* extracts against liver fibrogenesis.

Our in vivo results showed that *P. pavonica* and *J. rubens* extracts had hepatoprotective effects against acute liver injury. Meanwhile, *P. pavonica* and *J. rubens* have multiple pharmacological characteristics, including antioxidant and anti-inflammatory approaches. Therefore, we assumed that *P. pavonica* and *J. rubens* extracts would exert hepatoprotective approaches against acute liver injury by reducing oxidative stress and suppressing the inflammatory response, and the probable mechanism mediated by immune defense mechanisms including immune stimulation and immunomodulation.

### Assay of liver and kidney function

According to the obtained data of the current study, pre-and post-treatment of CCL_4_/mice with *P. pavonica* and *J. rubens* extracts led to a significant decrease in AST and ALT levels, dropping their elevated levels recorded in CCL_4_ intoxication and reversing them to a normal level comparable with control groups, indicating restoration of hepatic function and integrity and supporting the antioxidant hepatoprotective potentials of the seaweeds *P. pavonica* and *Jania rubens*, and this could be due to the fucoidan content of brown algae. Overall, the present results agree with research by Hira et al.^83^. , in which administration of brown seaweeds *Sargassum ilicifolium*, *Sargassum lanceolatum*, and *Sargassum swartzii* extracts in CCL_4_-induced liver damage mice preserved the level of hepatic enzymes AST and ALT low. Boshy et al.^61^. stated that fucoidan’s hepatoprotective efficacy against toxicity of CCl_4_ might be due to the downregulation of the inflammatory mediators and fucoidan’s antioxidant activities. *P. pavonica* and *J. rubens* treatment reduced serum levels of ALT and AST, implying a hepatoprotective impact against CCl_4_-induced liver damage, and pathological investigation verified this potential effect by successfully reducing the centrolobular necrosis area. Two pathogenic events were considered when trying to figure out the underlying mechanism: oxidative stress and inflammation because these two events usually act as the mechanisms for acute liver damage^81,84^. Oxidative stress-induced fibrosis occurs because of the excessive generation of ROS and its consequent hepatocyte damage by membrane lipid peroxidation^85^. CCL_4_-derived ROS results in hepatocyte membrane injury and leak of the hepatic enzymes into the circulation, which was confirmed by elevating the concentrations of serum ALT and AST^86^.

CCl_4_ at overdoses causes major hepatic lesions with large elevations in transaminases followed by liver and kidney damage^87,88^. Karakus et al.^89^. stated that acute liver injury affects kidney function and increases serum urea and creatinine levels. Importantly, our results revealed that intraperitoneal pre- and post-treatment of CCL_4_/mice with *P. pavonica* extracts showed the best results in significantly decreasing the level of serum urea creatinine compared to the naïve CCL_4_/mice control group. These results agree with Hira et al.^83^. , who revealed that rats treated with *Sargassum ilicifolium* and *Sargassum swartzii* extracts decreased the raised concentrations of renal function markers urea and creatinine in the CCL_4_-induced liver injury rats. This decrease may be owing to reduced oxidative stress or elevated excretion of hepato-toxicants outside the body. As a result, *P. pavonica* extract can be a challenged nephroprotective agent that could suppress the induction and progress of nephrocellular damage in association with its antioxidant efficacy. This efficacy might be owing to the antioxidant potentiation of its active antioxidant components.

### Histopathological examination

For further estimation of the hepatoprotective impacts of *P. pavonica* and *J. rubens* extracts in liver inflammation, a liver histopathology investigation was achieved to show the degree of the hepatic injury. After CCl_4_ intoxication in mice, hepatocyte necrosis was apparent in the central lobular areas of the liver section. However, pre- and post-treatment of CCL_4_/mice with *P. pavonica* and *J. rubens* extracts efficiently alleviated hepatotoxin-induced centrolobular necrosis. Significantly, all CCL_4_-induced histological changes were ameliorated by *P. pavonica* and *J. rubens* extracts. These findings support the results obtained from the current study’s immunomodulatory, anti-inflammatory, and antioxidant assays and strengthen the *P. pavonica* and *Jania rubens* extracts were able to minimize the deleterious effects of liver inflammation. Histological analyses of the *P. pavonica* and *J. rubens* extracts-treated mice groups exhibited reduced inflammatory process and suppressed liver necrosis and fibrosis. Chemically induced liver hepatotoxicity leads to the immediate production of ROS, resulting in lipid peroxidation and, consequently, membrane injury. Stimulated Kupffer cell generates toxic metabolites, including reactive oxygen intermediates and inflammatory cytokines, which are associated with the deterioration of hepatic parenchymal cells^71,73^. Our histological investigations agree with those of Barros-Gomes et al.^90^. and Cao et al.^91^. , who assessed the protective role of the *Gracilaria birdiae* algae in the liver of hepato-toxin-treated mice.

Additionally, treatment with brown seaweed *Padina boergesenii* extract inhibited hepato-toxin, triggering morphological changes in the liver, such as edematous hepatocytes. It displayed fewer cytoplasmic degeneration, fewer inflammatory infiltrations, and no necrosis^92^. Administration of the *P. pavonica* and *J. rubens* extracts could improve CCL_4_-induced histopathological alterations via changes in liver marker enzymes. The hepatoprotective action of *P. pavonica* and *J. rubens* extracts may be primarily due to the effects of their antioxidant-sulfated polysaccharides^93^.

The In vitro half maximal inhibitory concentration (IC50) of methanol extract of *P. pavonia* and *J. rubens* on hepatoblastoma cell line (HepG2) cell line after 24 h were estimated at 613 µg/ml and 475 µg/ml for, respectively. Additionally, In vivo half maximal lethal dose (LD) values of the methanol extract of P. pavonia and J. rubens were estimated at 1150 µg /kg and 1074 µg /kg, respectively^38^.

Brown seaweed extracts exhibit relatively high levels of chlorophyll, carotenoids, fucoxanthin polyphenol phlorotannins that have been shown to possess a range of bioactive properties, including anti-oxidant, anti-inflammatory, anti-allergic, anti-cancer, anti-obesity, and neuroprotective activities^35^.

The utilization of seaweed extracts as candidates for anti-inflammatory and anti-cancer therapies presents several drawbacks and challenges, including issues related to low bioavailability and chemical stability. The extensive side effects associated with these extracts can significantly hinder their therapeutic efficacy. Consequently, the quest of low-toxicity marine-derived pharmaceuticals aimed at preventing and treating inflammation and cancer has emerged as a key focus area for researchers^94^. Despite comprehensive investigations into the anti-inflammatory and anti-cancer properties of seaweed extracts, the application of fucoidan—an active component found in many seaweeds—as a therapeutic agent or drug delivery system has encountered notable obstacles. Primarily, the high cost and limited availability of pure fucoidan, a natural polymer sourced from seaweed, pose significant challenges in obtaining adequate quantities for research purposes^95^. Additionally, the extraction and purification processes are complex and labor-intensive. Furthermore, the structural complexity of these marine natural products complicates their manipulation and the development of cost-effective synthetic alternatives^96^. Another significant hurdle is the biological activity and selectivity of seaweeds natural products, as their origin from marine species rather than mammals may lead to undesirable biological responses in humans^97^. Lastly, ongoing safety assessments and biodegradability tests are crucial, as, despite the long-standing use of seaweed-derived bioactive components in food and dietary supplements, there remains a need to evaluate their safety and potential hepatotoxicity.

## Conclusion

Based on the above findings, our studies indicated that *P. pavonica* and *J. rubens* extracts could be potential candidates for chemical liver injury treatment by enhancing the immune and anti-inflammatory systems affecting hepatic inflammation and fibrogenesis progression. The hepatoprotective effects of *P. pavonica* and *J. rubens* may be mediated through their immune stimulation, immunomodulatory, anti-inflammatory, and antioxidant potentials. Additionally, *P. pavonica* and *J. rubens* extracts may be recommended as a potential natural therapy to cure, delay, or prevent conditions associated with inflammation or immune system disorders. Therefore, in another future study, we must search for their therapeutic potential in humans.

## Data Availability

The datasets used and/or analyzed during the current study are available from the corresponding author upon reasonable request.

## Abbreviations

CCL_4_: Carbon tetrachloride
WBCs: White blood cells
CRP: C-reactive protein
MDA: Malondialdehyde
AST: Aspartate aminotransferase
ALT: Alanine aminotransferase
NK: Natural killer
TNF-α: Tumor necrosis factor-alpha
IL-6: Interleukin 6
GSH: Antioxidant enzymes glutathione
